# Parkinson’s disease protein DJ-1 regulates ATP synthase protein components to increase neuronal process outgrowth

**DOI:** 10.1038/s41419-019-1679-x

**Published:** 2019-06-13

**Authors:** Rongmin Chen, Han-A Park, Nelli Mnatsakanyan, Yulong Niu, Pawel Licznerski, Jing Wu, Paige Miranda, Morven Graham, Jack Tang, Agnita J. W. Boon, Giovanni Cossu, Wim Mandemakers, Vincenzo Bonifati, Peter J. S. Smith, Kambiz N. Alavian, Elizabeth A. Jonas

**Affiliations:** 1 Department of Internal Medicine (Endocrinology), Yale University, New Haven, CT USA; 2 Department of Human Nutrition and Hospitality Management, University of Alabama, Tuscaloosa, AL USA; 3 Department of Cell Biology, Yale University, New Haven, CT USA; 4 Department of Neurology, Erasmus MC, Rotterdam, The Netherlands; 5 Neurology Service and Stroke Unit, Brotzu General Hospital, Cagliari, Italy; 6 Department of Clinical Genetics, Erasmus MC, Rotterdam, The Netherlands; 7 Institute of Life Sciences, University of Southampton, Southampton, England; 8 Marine Biological Laboratory, Woods Hole, MA USA; 9 Division of Brain Sciences, Department of Medicine, Imperial College, London, UK; 10 Department of Neuroscience, Yale University, New Haven, CT USA

**Keywords:** Mechanisms of disease, Diseases of the nervous system

## Abstract

Familial Parkinson’s disease (PD) protein DJ-1 mutations are linked to early onset PD. We have found that DJ-1 binds directly to the F_1_F_O_ ATP synthase β subunit. DJ-1’s interaction with the β subunit decreased mitochondrial uncoupling and enhanced ATP production efficiency while in contrast mutations in DJ-1 or DJ-1 knockout increased mitochondrial uncoupling, and depolarized neuronal mitochondria. In mesencephalic DJ-1 KO cultures, there was a progressive loss of neuronal process extension. This was ameliorated by a pharmacological reagent, dexpramipexole, that binds to ATP synthase, closing a mitochondrial inner membrane leak and enhancing ATP synthase efficiency. ATP synthase c-subunit can form an uncoupling channel; we measured, therefore, ATP synthase F_1_ (β subunit) and c-subunit protein levels. We found that ATP synthase β subunit protein level in the DJ-1 KO neurons was approximately half that found in their wild-type counterparts, comprising a severe defect in ATP synthase stoichiometry and unmasking c-subunit. We suggest that DJ-1 enhances dopaminergic cell metabolism and growth by its regulation of ATP synthase protein components.

## Introduction

Recent findings in the study of Parkinson’s disease (PD) have provided a unifying hypothesis to the apparently disparate functions of the proteins affected by familial genetic mutations. More specifically, many of the proteins encoded by these genes are necessary for normal mitochondrial function and protein trafficking, and show increased expression in tissues with high energy demands and frequent bouts of oxidative stress^1–7^. Rare but important mutations in the gene encoding DJ-1 are found in about 1% of familial cases of PD. DJ-1 is a peptidase C56 family protein with known and uncharacterized cellular functions^8^. Despite the creation of a DJ-1 null mouse^9^, the functions of DJ-1 are incompletely understood. DJ-1 mutant animals show increased sensitivity to neuronal toxins, and in different species DJ-1 is required for normal life span, motor function, and neuronal resistance to oxidative damage^10–12^. Defects in DJ-1 alter mitochondrial morphology and function^13^. DJ-1 translocates to mitochondria from the cytosol in response to mitochondrial stress^14,15^, suggesting that DJ-1 may assist Parkin and PINK1 in protein trafficking. DJ-1 also regulates mitochondrial metabolism since DJ-1 mutant cells have impaired ATP production and abnormal respiration^11^. Recent reports have emphasized that DJ-1 mutant mitochondria are sensitive to mitochondrial permeability transition (PT)^16^, one hallmark of cell death, and DJ-1 deficient mitochondria demonstrate abnormally high state 4 respiration, indicative of a leaky mitochondrial inner membrane^11^.

We recently described that a leak channel that decreases the efficiency of the ATP synthase is located within the c-subunit of the membrane-bound portion of the ATP synthase. We and others have suggested that an ATP synthase leak conductance forms a pathological mitochondrial permeability transition pore (mPTP)^17–22^ and that interaction of PD proteins may affect opening of the pore^22^. We described that the purified ATP synthase c-subunit ring forms channels in artificial bilayers, and that the channel is regulated by a complex of proteins forming and interacting with the F_1_ or enzymatic portion of the ATP synthase. Reports have shown that regulators within the F_1_ include the subunits β and OSCP, the latter of which binds cyclophilin D, an important regulator of mPTP opening and the target of the mPTP inhibitor cyclosporine A^20,23^. We have also shown that the antiapoptotic protein Bcl-xL, which binds the β subunit of F_1_ ATP synthase, and dexpramipexole (Dex), which binds the OSCP/b subunits, enhance the relative closure of the leak channel^24–26^.

A normal function of the leak channel is to adjust the level of uncoupling of oxidative phosphorylation to enhance or decrease metabolic efficiency. We now find that DJ-1 binds to the ATP synthase β subunit, influencing the probability of closure of the ATP synthase leak channel. DJ-1 also regulates the level of β subunit protein in mitochondria, ensuring the proper stoichiometric relationship of β subunit to c-subunit. This relationship is required for normal ATP synthase function. Thus, we find that DJ-1 regulates normal ATP synthase function and stoichiometry to enhance growth of neuronal processes, protect cells from death, and promote oxidative metabolic efficiency.

## Results

### DJ-1 is localized to the ATP synthasome and interacts directly with ATP synthase β subunit

**Fig. 1:**
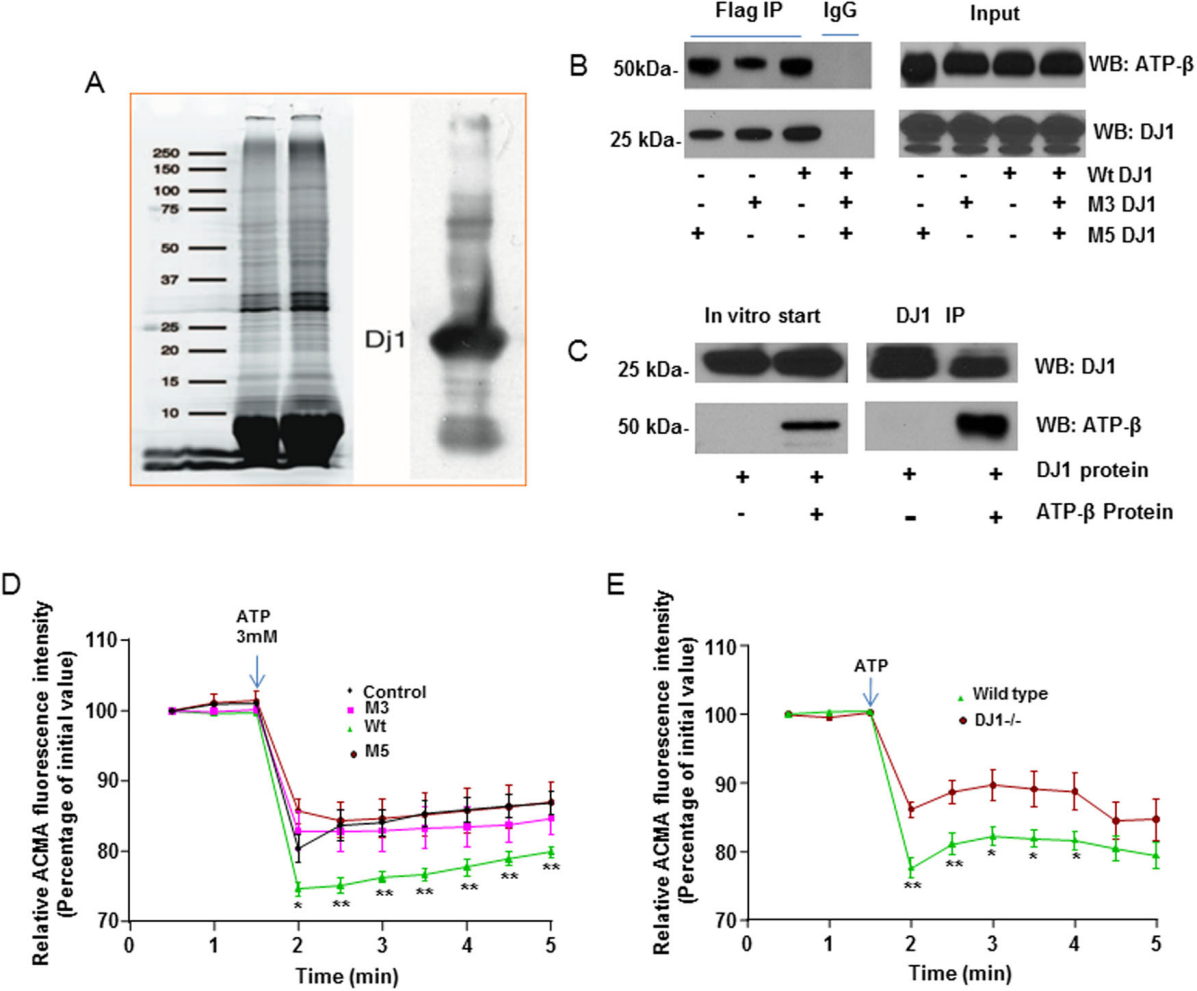
DJ-1 is localized to sub mitochondrial membrane vesicles enriched in ATP synthase (SMVs). **a** Coomassie staining of the protein bands present in submitochondrial vesicles (SMVs) is shown in the left panel. The right panel shows an immunoblot using an anti-DJ-1 antibody against solubilized protein from SMVs. The blot confirms that DJ-1 is present in the SMV subcellular fraction. **b** WT and mutant DJ-1 immunolocalize with ATP synthase β-subunit. Extracts from HEK293 cells expressing flag-tagged WT or mutant DJ-1 were prepared for coimmunoprecipitation analysis with anti-FLAG antibody. Proteins were revealed with the indicated antibodies in western blots. Note that endogenous ATP synthase β-subunit is copurified with WT DJ-1 and also with mutant DJ-1. **c** Purified WT and mutant DJ-1 immunolocalize with ATP synthase β-subunit in vitro. Purified flag-ATP synthase β-subunit protein was incubated with purified DJ-1. The mixture was immuno-precipitated with anti-DJ-1 antibody. The protein interaction was revealed by western blotting with the indicated antibodies. **d** The ability of SMVs to sequester H^+^ is improved by recombinant WT DJ-1 protein. Shown are ACMA (H^+^ indicator) fluorescence intensity changes induced in response to ATP in the presence of rat SMVs. A decrease in indicator intensity represents sequestration of H^+^ into the lumen of SMVs. Data were acquired every 30 s for 5 min in the presence of BSA control protein or recombinant protein (all proteins were added at 10 µg/100 µl final). For WT DJ-1 (*n* = 6 samples), M3 DJ-1 (*n* = 8) or M5 DJ-1 (*n* = 8). WT DJ-1 protein maximizes the ability of SMVs to sequester H^+^ whereas mutant DJ-1 proteins have no effect. **p < 0.01, *p < 0.05, recombinant DJ-1 protein versus BSA control (*n* = 6). Paired, two-tailed *t*-test. Error bars represent mean ± SEM. **e** The ability of SMVs to sequester H^+^ is compromised in DJ-1^−/−^ SMVs. WT (*n* = 7) and DJ-1^−/−^ (*n* = 6). **p < 0.01, *p < 0.05, Error bars represent mean ± SEM

In looking for ATP synthase binding partners or modulators of the ATP synthase leak channel, we isolated submitochondrial vesicles (SMVs) that are relatively enriched in ATP synthase^24,27,28^. Liquid chromatography mass spectrometry analysis of the proteins from SMVs revealed the presence of DJ-1. To confirm this, we performed immunoblots of SMVs with anti-DJ-1 antibodies and demonstrated the presence of DJ-1 (Fig. 1a). In previous reports, we showed that the anti-cell-death protein Bcl-xL was bound to the β subunit of the ATP synthase and that it regulates inner mitochondrial membrane leak to enhance cellular metabolism^24,26^. To determine if DJ-1 were also bound to the β subunit, we immunoprecipitated FLAG-tagged DJ-1 from HEK-293T cells using beads conjugated to an anti-FLAG antibody (Fig. S1a); this revealed colocalization of DJ-1 and the β subunit (Fig. 1b). Since the interaction was robust, we tested the interaction of purified WT DJ-1 with ATP synthase β subunit in vitro (Fig. 1c). Purified recombinant FLAG-tagged ATP synthase β subunit protein was incubated with purified recombinant DJ-1, and the mixture was immunoprecipitated with anti-DJ-1 antibodies. This demonstrated that the two proteins bind to each other directly and do not require other binding partners for their interaction. We also found that DJ-1 coimmunoprecipitates with Bcl-xL (Fig. S1b), suggesting that the two proteins could regulate cellular metabolism by working together in a complex. To test if DJ-1, like Bcl-xL, is present in mitochondrial inner membranes in the brain, we performed immunoelectron microscopy. We found that DJ-1 localizes both to outer membrane and to inner membrane cristae in 9/12 micrographs.

### Mutant DJ-1 proteins fail to enhance ATP synthase function

ATP synthase produces ATP in mitochondria. To study the function of WT DJ-1 and loss of function DJ-1 mutations in mitochondrial ATP production, we expressed DNA constructs for known PD-associated DJ-1 mutations (M3 = A104T; M4 = D149A; M5 = L166P;^12^ and for WT DJ-1 in HEK 293T cells; we also purified the proteins). Surprisingly we found that all mutant DJ-1 proteins bound effectively to both the ATP synthase β subunit and to Bcl-xL (Fig. 1b and S1b). We also noted that the C-terminal 60 amino acid deletion in DJ-1 does not affect its binding to the ATP synthase β subunit (Fig S1c). Therefore, we determined if binding was sufficient to confer normal ATP synthase enzymatic function. To assess if DJ-1 binding to the β subunit were required for normal inner membrane H^+^ ion sequestration, we performed a 9-amino-6-chloro-2-methoxyacridine (ACMA) assay. SMVs purified from rat brain were incubated with the H^+^ fluorescent indicator ACMA and exposed to ATP^24,29^. ATP hydrolysis in this assay produces sequestration of H^+^ ions into the lumen of the SMVs, and an accompanying decrease in bath fluorescence of the SMV-excluded H^+^ indicator. The steady state response to ATP was enhanced by the addition of WT DJ-1 protein to the bath. In contrast, the addition of mutant proteins failed to enhance the steady state level of H^+^ ion sequestration (Fig. 1a and S1d). DJ-1 deficient SMVs isolated from DJ-1^−/−^ mouse brain also failed to sequester H^+^ normally in response to ATP hydrolysis (Fig. 1e).

**Fig. 2:**
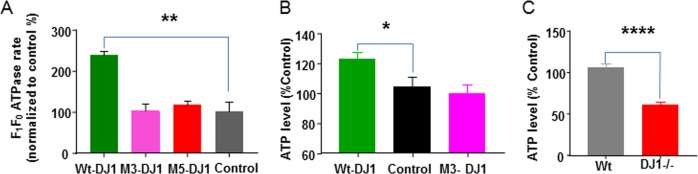
DJ-1 is required for optimal efficiency of ATP synthase enzyme activity. **a** Recombinant WT DJ-1 protein, but not M3 and M5 mutant proteins, increases ATP hydrolysis enzymatic rate in an assay where ATP level is not rate-limiting (see methods). Recombinant proteins were added to the wells during the assay (*N* = 4 for each assay group; **p < 0.01. Error bars represent mean ± SEM; unpaired, two-tailed t-test.). **b** WT DJ-1, but not mutant DJ-1 (M3), increases endogenous ATP levels when overexpressed in HEK293 cells (*p < 0.05, *n* = 7, Error bars represent mean ± SEM). **c** The endogenous ATP level in DJ-1^−/−^ midbrain culture is significantly lower than that of WT midbrain cultures. Mean ± SEM is shown. *N* = 72 wells from three independent paired primary cultures (****p < 0.0001)

An improvement in H^+^ sequestration in the ACMA assay may indicate an increase in ATPase rate, a decrease in inner membrane leak or both. To differentiate between these effects, we measured ATPase rate and leak directly using different techniques. ATPase activity was assessed in an ATP regenerating system where ATP concentration is not rate-limiting (Fig. 2a and S2). WT DJ-1 protein markedly enhanced enzymatic rate while mutant forms of DJ-1 protein did not enhance ATPase activity over control. To test if DJ-1 or mutants regulated the availability of ATP, ATP levels were measured in DJ-1 overexpressing HEK 293 T cells and primary midbrain cultures. ATP levels were found to be significantly higher when WT DJ-1, but not mutant DJ-1, was overexpressed (Fig. 2b). ATP levels were lower in DJ-1^−/−^ midbrain cultures compared to those of WT controls (Fig. 2c).

### Mutant DJ-1 proteins fail to close an inner mitochondrial membrane leak

**Fig. 3:**
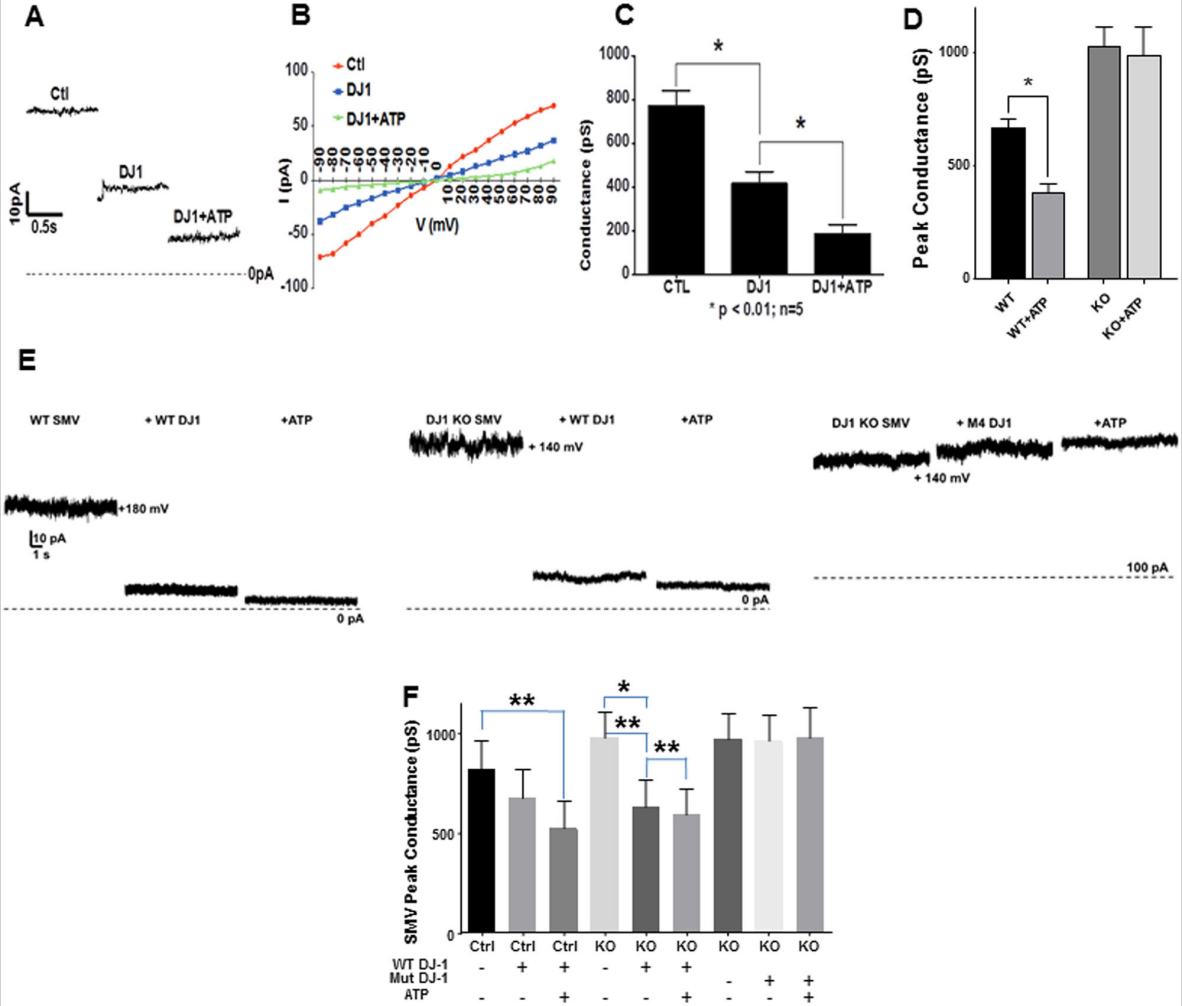
DJ-1 is required to optimally decrease mitochondrial inner membrane leak conductance. **a** Recombinant WT DJ-1 protein decreases SMV membrane conductance in patch clamp recordings. Sequential patch recordings before and after addition of 10 µg/ml DJ-1 recombinant protein to the bath and after exposure to DJ-1 + 1 mM ATP. **b** Current-voltage relationship is linear for the effects of recombinant DJ-1 protein and ATP on total SMV membrane conductance. **c** Peak membrane conductance (*G* = *V*/ΔI, where *G* is the conductance in picosiemens, *V* is the membrane holding voltage in millivolts and ΔI is the peak membrane current in pico-amperes minus the baseline current in pico-amperes) was measured in patch clamp recordings of SMVs before and after addition of the indicated agents (*N* = 5; *p < 0.01). **d** The ability of 1 mM ATP to decrease peak membrane conductance is compromised in DJ-1^−/−^ SMVs. WT and DJ-1^−/−^ SMVs were prepared from 7-week-old female mice. WT patch clamp recordings (*N* = 7), DJ-1^−/−^ (*N* = 4; *p < 0.05). **e** The ability of mutant DJ-1 recombinant protein but not WT DJ-1 protein to decrease membrane conductance is compromised in DJ-1^−/−^ SMVs. Shown are example SMV patch clamp recordings from (left to right) WT, DJ-1^−/−^ before, and after addition of the indicated agents. **f** Group data for the patch clamp recordings shown in **e** (Paired recordings *N* = 12 CTL, *N* = 14 DJ-1^−/−^ with added recombinant WT DJ-1 protein, *N* = 8 DJ-1^−/−^ with added recombinant mutant (M4) DJ-1 protein (10 µg/ml); ***p = 0.0004 by overall ANOVA)

The ATPase assays suggested that WT DJ-1 enhances ATPase enzymatic rate; these assays, however, do not specifically test whether DJ-1 prevents mitochondrial inner membrane leak. Therefore, to determine whether the effect of DJ-1 on H^+^ ion sequestration rate was due to a decrease in the inner membrane leak produced by DJ-1, we performed patch clamp recordings of rat brain SMVs. WT control recordings showed significantly higher ion channel conductance at all voltages recorded compared to SMVs exposed to recombinant WT DJ-1 protein, suggesting that addition of extra, exogenous DJ-1 further decreases leak channel activity even in WT SMVs (Fig. 3a–c). Conductance was further decreased by addition of ATP to the bath, consistent with previously reported findings^17,24^. WT mouse SMVs also responded with a rapid decrease in conductance after the addition of ATP, while DJ-1^−/−^ SMVs failed to respond to ATP, suggesting that the ability of ATP to alter the inner membrane ion channel conductance is dependent on the presence of DJ-1 (Fig. 3d). To test this finding further, we recorded DJ-1^−/−^ SMVs and added either recombinant WT or mutant DJ-1 protein to the bath during recordings. The addition of WT DJ-1 decreased conductance of the patch in both WT mouse brain SMVs and DJ-1^−/−^ SMVs, so much so that there was little additional effect of ATP on inner membrane conductance, whereas the addition of mutant protein samples had no effect (Fig. 3e, f). ATP also failed to decrease conductance in DJ-1^−/−^ SMVs exposed only to mutant DJ-1 protein. This latter finding confirmed that WT DJ-1 and ATP are required for maximally decreasing leak conductance (Fig. 3e, f).

**Fig. 4:**
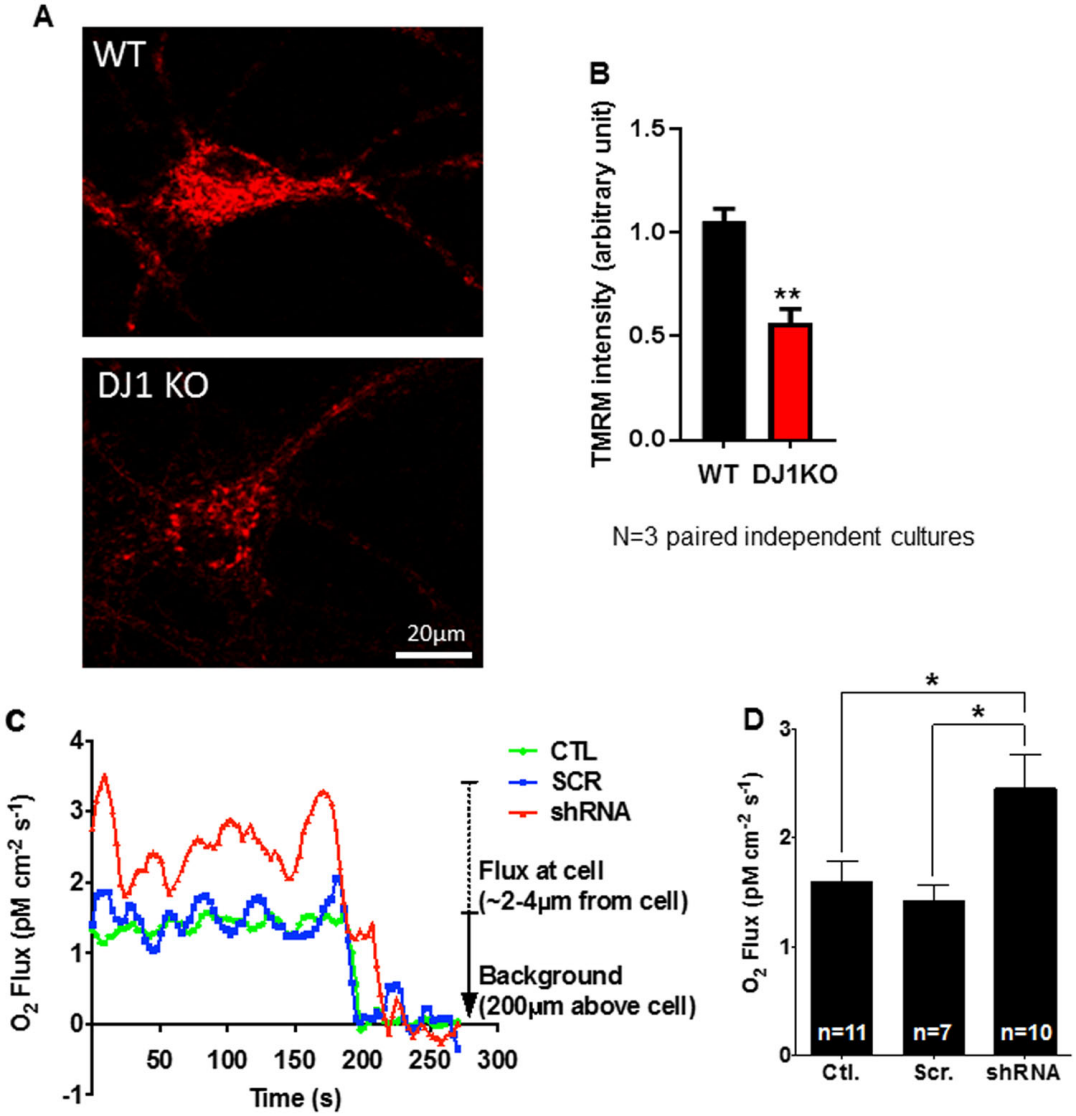
DJ-1^−/−^ neurons have reduced mitochondrial membrane potential. **a** Representative images of mitochondrial membrane potential indicator TMRM of WT and DJ-1^−/−^ neurons. **b** Group data for images shown in **a**. TMRM fluorescence intensity was calculated by drawing a large region of interest that encompassed the entire soma (*n* = 45 WT neurons and *n* = 43 DJ^−/−^ neurons from three independent paired midbrain cultures, **p = 0.005964). **c** Presence of DJ-1 reduces oxygen consumption of single hippocampal neurons. Panel shows representative traces of single neuron oxygen electrode recordings performed in WT neurons or WT neurons expressing scrambled or DJ-1 shRNA. **d** Group data of peak oxygen consumption for single neurons expressing the indicated constructs (*N* = 7–11 cells each; *p = 0.0136)

The decrease in inner membrane leak and the increase in ATPase enzyme activity may prevent a loss of inner membrane potential caused by metabolic activities or by a pathological inner membrane leak. In support of this, mitochondrial membrane potential was found to be relatively hyperpolarized in normal WT neurons compared to neurons lacking DJ-1 (Fig. 4a, b and S3). Hyperpolarization of mitochondrial membrane potential could accompany an increase in efficiency of ATP production associated with less substrate uptake per ATP molecule produced^24^. We had already found an increase in ATP levels in the neuronal cultures (Fig. 2). We therefore measured oxygen consumption in single neurons. Single neuron oxygen sensors deliver real time oxygen flux detection with high spatial resolution^24,25,30–32^, allowing the interrogation of single cells using a 1–2 µm diameter tip held within 1 µm of the cell surface. Using the sensor, we found that oxygen consumption by single WT neurons was significantly lower than that in neurons depleted of DJ-1 by shRNA (Fig. 4c, d), suggesting that the low inner membrane leak increases ATP production per unit oxygen consumed.

### DJ-1 enhances growth of neuronal processes

**Fig. 5:**
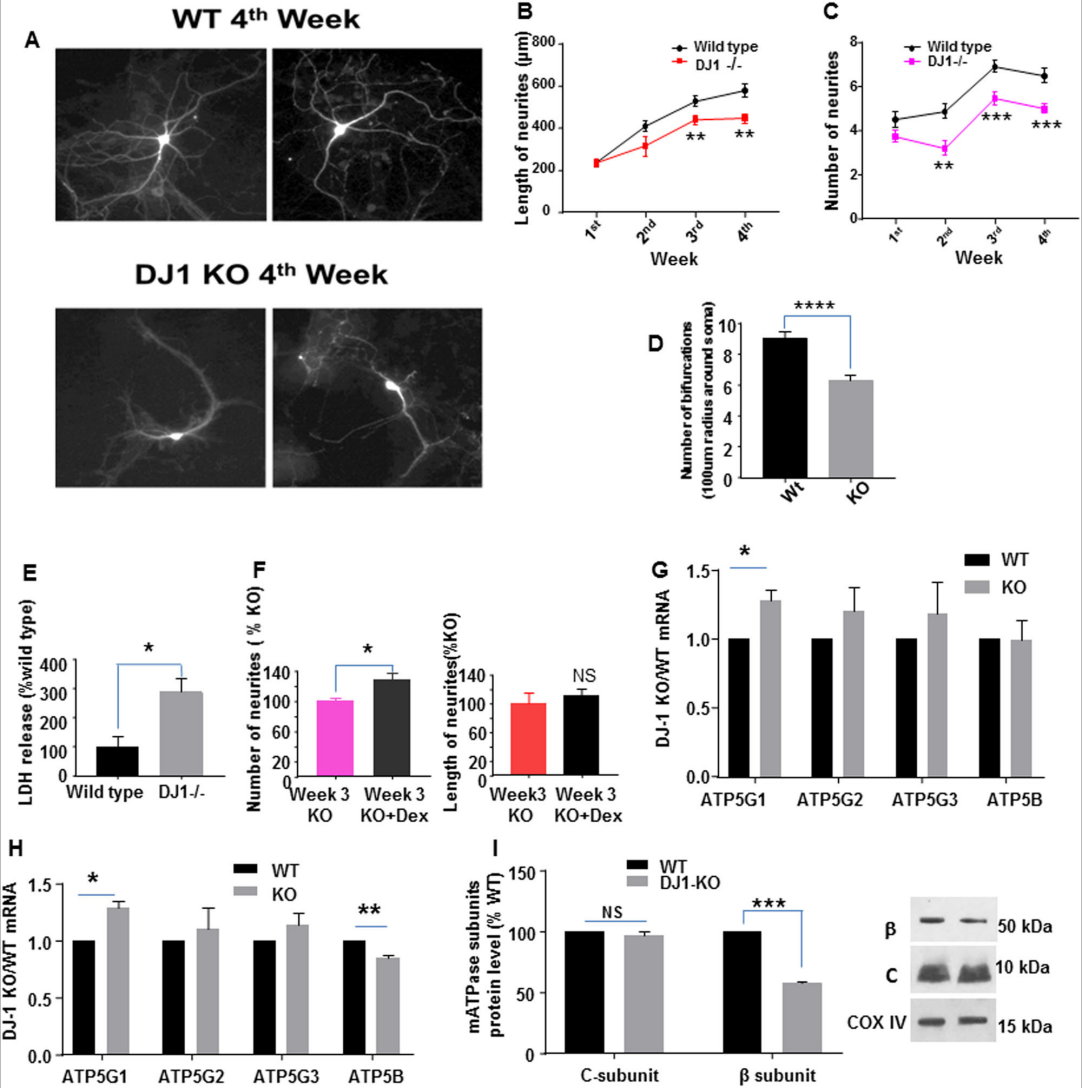
DJ-1 depletion inhibits neurite outgrowth of dopaminergic neurons. a Representative tyrosine hydroxylase (TH) antibody-stained neurons. **b** The length of neurites at four different time points in vitro (First week: WT (*n* = 26 TH+ neurons), KO (*n* = 20); second week: WT (*n* = 25), KO (*n* = 16); third week: WT (*n* = 41), KO (*n* = 32), **p = 0.0091; fourth week: WT (*n* = 22), KO (*n* = 32), **p = 0.0012). **c** The number of neurites extending directly from the soma at four different time points in vitro (First week: WT(*n* = 26), KO (*n* = 20); second week: WT (*n* = 25), KO (*n* = 16), **p = 0.0022; third week: WT (*n* = 41), KO (*n* = 32), ***p = 0.00073; fourth week: WT (*n* = 22), KO (*n* = 32), ***p = 0.00026). **d** The number of bifurcations at the third week (*n* = 20 WT TH+ neurons and *n* = 20 DJ-1^−/−^ TH + neurons, ****p < 0.0001). **e** LDH release (into the medium) at the third week of culture (*n* = 3 cultures, *p = 0.0052). **f** Neurite outgrowth of dopaminergic neurons is improved by 30 µM Dex. Number of neurites extending directly from the soma at the third week (**Left**). Length of neurites at the third week (**Right**). The length of untreated KO dopaminergic neurites and the number of untreated KO dopaminergic neurites are set as 100%, Dex treatment affects the number of neurites arising from the soma. Mean ± SEM (*n* = 3 independent midbrain cultures, *p = 0.018 for neurite number, at least 10 cells counted per group in each culture). **g** ATP synthase c-subunit 5G1 mRNA is increased in DJ-1^−/−^ compared to WT midbrain cultures. The mRNA level in WT cells was set as one, mean ± SEM (*n* = 3 independent cultures, *p = 0.027 for *ATP5G1*). **h** mRNA expression of ATP5G and ATP5B in 7-month-old mouse midbrain tissue. The data are quantified by qRT-PCR. The mRNA level in WT was set as one, mean ± SEM (*n* = 3 for WT mouse and *n* = 3 for DJ-1^−/−^ mouse, *P = 0.0108 for ATP5G1 and **p = 0.0027 for ATP5B). **i** Mitochondrial protein expression of ATPase c-subunit is unchanged (**Left)**, but mitochondrial protein expression of ATPase β subunit is decreased in DJ-1 KO brain (**Right**) of 7–8-month-old animals. Protein level in WT was set as 100%. ***p = 0.0002 for ATP synthase β subunit level

Neurons experiencing metabolic derangements may be placed at risk for degeneration, developmental defects, or defects in growth or branching of neuritic processes. We found that during 4 weeks in culture, neurites of midbrain dopaminergic neurons (stained with anti-TH antibody; Fig. S4a) isolated from DJ-1^−/−^ mice failed to reach the same length as WT neurites and there was a decrease of neurite numbers at initiation sites on the neuronal somata (Fig. 5a–c). The number of branch points of each neurite was also reduced (Fig. 5d). These growth deficiencies were associated with chronic cell death in the cultures as measured by lactate dehydrogenase (LDH) release into the medium by the third week (Fig. 5e). Enhanced death rate was most likely related to the abnormal metabolism produced by the absence of DJ-1. To determine if closing the inner membrane leak would improve functional outcomes, we treated with the pharmacological reagent Dex, that we have shown previously to decrease inner mitochondrial membrane conductance and ameliorate cell death^25,33,34^. Dex effectively rescued the loss of neurites at the cell soma in dopaminergic neurons cultured chronically over 3–4 weeks, although neurite length was not significantly improved (Fig. 5f and S4b), suggesting that attempted pharmacological improvement in inner membrane coupling was only partially successful in preventing loss of neurites and neurite growth in DJ-1 deficient cultures.

We had suggested previously that the ATP synthase c-subunit leak channel is gated by interaction with components of the F_1_^17^. If some F_1_ components were lost in the DJ-1 deficient cultures, this would eliminate the binding site for common reagents used to enhance inner membrane coupling like Dex^20,23^. To determine if the metabolic and growth defects produced by the lack of DJ-1 were related to relative loss of F_1_ components compared to c-subunit, we examined c-subunit and β subunit mRNA in mesencephalic cell culture by qPCR. The main isoform of c-subunit in neurons, ATP5G1 mRNA, was significantly increased in DJ-1 deficient neurons compared to WT neurons, while levels of β subunit mRNA were unchanged (Fig. 5g). This was also the case in aging mouse brain where ATP5G1 (c-subunit) mRNA was again found to be significantly increased in DJ-1 deficient brain tissue while ATP5B (β subunit) mRNA was reduced (Fig. 5h). Immunoblots of aging brain mitochondria revealed that β subunit protein level was substantially reduced, while c-subunit protein level was relatively unaffected (Fig. 5i), suggesting a change in stoichiometry within the ATP synthase proteome that would predispose mitochondria to a leaky inner membrane.

## Discussion

### DJ-1 binds to the β subunit to affect ATP synthase enzyme function and inner mitochondrial membrane permeability

We show in isolated neurons, HEK cells and isolated mitochondria that mutation or loss of DJ-1 cause a decrease in ATP synthase enzymatic rate, a decrease in cellular ATP levels, loss of mitochondrial inner membrane potential and development of a large leak in the mitochondrial inner membrane. These findings are accompanied by decreased protein levels of ATP synthase β subunit and a decrease in the ratio of β subunit mRNA and protein to those of c-subunit. We suggest that this promotes ATP synthase leak channel opening. Our previous studies have shown that the c-subunit can form a large conductance channel under conditions of excitotoxic stress^17,35^, suggesting that the membrane portion of the ATP synthase constitutes the main pore-forming unit of the mPTP. We show here in patch clamp recordings that DJ-1 decreases the propensity for the mitochondrial inner membrane leak to open. Our findings of inner membrane depolarization in the absence of DJ-1 are supported by previous studies in the field that have suggested that loss of DJ-1 leads to inner membrane depolarization, PT^16^, and oxidative stress^36^. In a role for DJ-1 that has never been previously suggested, we find that the binding of DJ-1 to the β subunit increases ATPase activity, the level of ATP in cells, and decreases oxygen uptake, enhancing inner membrane coupling, and metabolic efficiency of oxidative phosphorylation. In addition, the correct stoichiometry between the F_1_ and c-subunit, regulated by the presence of WT DJ-1, appears to be required for full efficiency in ATP synthase function.

### DJ-1 enhances β subunit amount in mitochondria

DJ-1 has been described to have multiple functions, particularly as a modulator of oxidative stress, in part by directly protecting cells from toxic glycolytic products by becoming oxidized itself^37^. DJ-1 also acts as a coactivator of various transcription factors including the androgen receptor, p53, PSF, Nrf2, SREBP, and RREB1 and the LDL receptor^38–40^. In this role, DJ-1 upregulates signaling pathways involved in the expression of genes that combat oxidative stress and it enhances pathways regulating cell survival and growth^10^.

Recent reports have also suggested that DJ-1 acts as a chaperone or chaperone regulator; increasing DJ-1 expression increases levels of Hsp70, preventing aggregation of alpha synuclein^41^. WT as well as mutant DJ-1 have been found to associate with Hsp70, mitochondrial Hsp70/Grp75 (mortalin), and the cochaperone C-terminus of Hsp70 interacting protein. DJ-1 translocates into mitochondria upon oxidative stress accompanied by other chaperones suggesting that it works together with these chaperones^42^. Further evidence of its chaperone function is that overexpression of DJ-1 protects neurons from alpha synuclein aggregation by increasing the expression of LAMP-2A, suggesting that chaperone mediated autophagic function is enhanced by DJ-1^43,44^. Nevertheless, despite known mitochondrial localization by DJ-1, chaperone function of DJ-1 for ATP synthase subunits, as suggested by our study, has not been previously reported. We now find that DJ-1 is necessary for the proper complement of β subunit protein in mitochondria, but whether DJ-1 acts at the transcriptional level, protects β subunit mRNA, helps with translation of β subunit mRNA and/or helps translocate β subunit into the mitochondria or all of the above remains to be fully studied. We suggest that these functions may be key to regulate ATP synthase activity under stressful conditions or during aging.

### β subunit of ATP synthase forms a hub for regulation of metabolism

The ATP synthase β subunit and other components of the enzymatic F_1_ portion of the synthase respond to, and regulate, metabolic flexibility. ATP synthase F_1_ subunits work in a complex with regulators, including antiapoptotic proteins such as Bcl-xL. In mouse neurons with decreased levels of F_1_ components including OSCP and the β subunit, mitochondrial membrane potential is depolarized, accompanied by increased oxidative stress, early PT, low ATP levels and decreased synaptic function^45^. The same group also found low levels of F_1_ components in Alzheimer’s-diseased brains. We have previously reported that the anti-death protein Bcl-xL binds to the β subunit of the ATP synthase and enhances mitochondrial ATP production efficiency^24,26^. This leads to enhanced synapse formation and enhanced synaptic transmission^46,47^ as well as increased localization of mitochondria to synaptic sites through activation of mitochondrial fission^47,48^. Our current study suggests that in addition to its own effects on β subunit and ATP synthase function, DJ-1 also binds to Bcl-xL, perhaps enhancing Bcl-xL’s activity to increase ATP production, mitochondrial positioning, and neurite extension.

### Cell and axonal growth are affected by loss of DJ-1

Evidence suggests that normal neuronal development is dependent upon kinase pathways that assist in mitochondrial positioning at axonal branch points^49^. Mitochondrial morphological changes consistent with a loss of fusion/fission have been observed in neurons and patient cells with DJ-1 deficiency^50^. A recent study shows that Parkin KO neurons grown on astrocyte monolayers of the same genotype have a markedly decreased axonal arborization, increased basal oxygen consumption rate, and decreased ATP production, consistent with uncoupling^51^. These neurons also have reduced dopamine transporter, as a possible mechanism for decreased dopaminergic function in parkin deficient animals. Although no change in axonal arborization was found for DJ-1 neurons in that study, the authors acknowledged that increasing time in the culture might be necessary to detect changes. Indeed, our findings show that growth of neuronal processes over 4 weeks in culture is dependent upon DJ-1, and we show that marked slowing of growth, with accompanying neuronal loss, occurs over this time in DJ-1 lacking cells. Decreased neuronal process outgrowth was also observed in an animal model of DJ-1 loss on a C57B6 background. In that report, in the SNc, marked tyrosine hydroxylase (TH)^+^ neuronal loss was demonstrated and neurons clearly had shortened neuronal processes and process beading, with age dependent loss of process length and sprouting, decrease in DA-synthesizing TH^+^ striatal terminals and motor dysfunction^52^. In the DJ-1 KO neurons, our current findings suggest partial rescue of the lack of growth of the neurites with Dex, a reagent that we have shown increases mitochondrial inner membrane coupling by binding to ATP synthase F_1_. We found that the effect of Dex was only partial, perhaps because, in the absence of the full complement of β subunit, Dex is unable to fully close the leak^53^.

Our data suggest that DJ-1 is required for creating the normal stoichiometry of the ATP synthase and for assisting with positioning of ATP synthase β subunit to fully close the mitochondrial inner membrane leak. Therapeutic potential would therefore be provided by agents that enhance or mimic the functions of DJ-1 to position the β subunit, to synthesize β subunit, and to translocate β subunit into the inner membrane. Our study suggests a role for DJ-1 in transcriptional/translational regulation and chaperone function directed specifically at the ATP synthase β subunit.

## Methods

### Animals

WT mice and DJ-1^−/−^ mice were from The Jackson Laboratory (C57BL/6, Stock no: 000664; B6.Cg-Park7^tm1Shn^/J, Stock no: 006577) and the original DJ-1^−/−^ mouse was previously described^54^. Sprague-Dawley rats were from Charles River. All animals were maintained in a climate-controlled room and kept on a 12:12-h light–dark cycle with food and water available. All procedures were performed in accordance with the NIH Guide for the Care and Use of Laboratory Animals and approved by Yale University’s Institutional Animal Care and Use Committee (IACUC).

### Recombinant protein expression and purification

The human DJ-1 and ATP synthase β (ATP5B) constructs with Myc and DDK (Flag) tags on the C-terminus were from OriGene Technologies. Five different DJ-1 mutations were described in a previous study^12^, M1 (M26I), M2 (E64D), M3 (A104T), M4 (D149A), and M5 (L166P) were made from the original DJ-1 construct using a Quick-change Site-Directed Mutagenesis Kit (Agilent Technologies). HEK293 cells were cultured in 15 cm cell culture dishes in high glucose DMEM (10% v/v FBS, 100 U/ml penicillin, 100 μg/ml streptomycin). All the plasmid transfections were performed with Lipofectamine 2000 (Invitrogen). Recombinant proteins were purified using the EZview Red ANTI-FLAG M2 Affinity Gel (Sigma, F2426), according to the manufacturer’s protocol. The expression was verified by western blot analysis according to previously published protocols^17,55^.

### Western blot, Coimmunoprecipitation, and in vitro IP

Cells were lysed at 4 °C in extraction buffer (0.1 M NaCl, 20 mM HEPES pH 7.5, 1 mM EDTA, 5 mM NaF, 1 mM dithiothreitol, 0.3% Triton X-100, 5% glycerol, 0.25 mM phenylmethylsulfonyl fluoride, and complete protease inhibitor cocktail and phosphatase inhibitors). Homogenates were cleared by centrifugation (12,000 g, 10 min). Coimmunoprecipitation was performed according to previous techniques^55^. In vitro IP was performed using purified recombinant proteins instead of the cell lysate. The antibodies used in the study were as follows: Anti-Myc (Cell Signaling, #2276); Anti-DDK (Origene, TA50011); Anti-DJ-1(Santa Cruz, sc27006 and sc32874; Cell Signaling, 2134S); Anti-β-Actin, #4970); Anti-GAPDH (Santa Cruz, sc-32233); Anti-mATP5G1/2/3(abcam, ab180149); Anti-ATPB (abcam, ab14730), and Anti-Bcl-xL(Cell Signaling, #2764); Anti-Puromycin (3RH11) (Kerafast, EQ0001).

### Isolation of mitochondria

Mitochondria were isolated from human fibroblasts following QproteomeTM Mitochondria isolation kit manual (Qiagen, Cat.37612). In brief, cell pellets were harvested and washed once using 0.9% NaCl, then cells were lysed in buffer and the lysate was centrifuged at 1000 *g* for 10 min. The supernatant was saved as the cytosolic protein sample. Cell disruption was carried out using a 1 ml syringe with a blunt-ended needle in disruption buffer (provided in the kit) times^10^. The lysate supernatants were saved after centrifugation at 1000 *g* for 10 min. The pellet disruption step was repeated and the supernatants were combined. The mitochondrial pellets were collected after supernatants were centrifuged at 6000 *g* for 10 min. After washing with storage buffer and centrifuging at 6000 *g* for 20 min, the mitochondrial pellets were resuspended in lysis buffer as the mitochondrial protein sample.

### Isolation of SMVs from rodent brain

Rodent brain tissue (without cerebellum) was finely minced and homogenized in ice-cold isolation buffer [250 mM sucrose, 20 mM Hepes (pH 7.2), 1 mM EDTA, and 0.5% BSA]. After a centrifugation at 1500 g to pellet nuclear material, the supernatants were centrifuged at high-speed (16,000 × *g*) for 10 min at 4 °C. The crude pellets were resuspended in isolation buffer and a pressure of 1200 Psi was applied for 10 min, followed by rapid decompression. The pure mitochondrial fraction was then pelleted in a ficoll density gradient by centrifugation and washed with isolation buffer. Nonionic detergents (digitonin and Lubrol-PX) were used to further solubilize and stabilize membrane-bound protein complexes, and the submitochondrial SMVs were isolated by a final 2 h ultracentrifuge. Freshly prepared SMVs were quantitated using the Bradford protein assay and used in ACMA experiments and in the ATP regeneration system.

### ACMA assay

ACMA (Sigma A5806) fluorescence quenching was measured according to previously published methods^24^ with some modifications. In brief, 2 µM ACMA, 50 μg isolated rat brain F_1_F_O_ ATPase vesicles, and equal amount of either WT DJ-1 or mutant DJ-1 protein was used in each reaction. F_1_F_O_ ATPase vesicle suspensions were measured at 490 nm using a PerkinElmer VICTOR3 multilabel plate reader. Similar assays were performed on WT and DJ-1^−/−^ mice brains.

### Neuronal culture and studies of cultured neurons

WT and DJ-1^−/−^ midbrain (or cortical) cultures were prepared from embryos isolated from a pregnant female mouse at day-13 post coitum. Cells were dissociated and seeded on Poly-Lysine pre-treated culture dishes with neurobasal medium, supplemented with B-27, glutamine, antibiotics and 5% FBS (Invitrogen GIBCO Life Technologies, Carlsbad, CA, USA). After 2 hr incubation, primary cultures were maintained in neurobasal medium without FBS in 5% CO2 incubator at 37 °C for 3-4 weeks. Glia were not removed from the cultures. For cellular ATP levels, cells were plated into 96-well plates, and ATP levels were measured at DIV 14-16. For cell death, cells were plated into 12-well plates and LDH was measured at DIV 21-22. To measure neurite outgrowth, cells were plated into 6-well plates with cover glass, and fixed for immunocytochemistry (ICC) with anti-TH+ antibody (Pel Freez Biologicals, p40101-150) as described previously^55^. Images were captured using a Zeiss inverted fluorescent microscope. The number of neurites extending from the soma were counted and the longest extent of dendrites from tip to tip on opposite sides of the soma at 180° were measured using AxioVision 4.8. Numbers of bifurcations (within 100 µm radius of the soma) were counted in Image J^55^. For mitochondrial membrane potential, cortical cultures were plated into 6-well plates containing cover glasses, and confocal images were captured at DIV 21 after TMRM staining.

### ATP synthase activity

ATP synthase activity was measured using the ATP synthase Specific Activity Microplate Assay Kit (abcam, ab109716). ATP Synthase is immunocaptured within the wells. ATP hydrolysis results in production of ADP which is ultimately coupled to the oxidation of NADH to NAD+ that is then monitored as a decrease in absorbance at 340 nm. Fresh rat SMVs were adjusted to concentration 5.5 mg/mL, and then extracted in detergent buffer (provided with the kit) on ice for 30 min followed by centrifugation at 16,000 rpm for 20 min. Micro plates with 5 µl SMVs per well were kept at 4 °C overnight. Each well was rinsed twice with solution 1 and incubated with lipid mix for 40 min. An equal amount of WT DJ-1 or mutant DJ-1 protein was added into each reaction, and incubated for another 5 min. Reagent mix was added to the reaction and ATP synthase activity was measured at OD340 at 1 min intervals for 1–2 h.

### Electrophysiology

F_1_F_O_ ATPase vesicle (SMV) recordings were made by forming a giga-ohm seal onto SMVs in intracellular solution (120 mM KCl, 8 mM NaCl, 0.5 mM EGTA, and 10 mM HEPES, at pH 7.3) using an Axopatch 200B amplifier (Axon Instruments) at room temperature (22–25 °C). Recording electrodes were pulled from borosilicate glass capillaries (World Precision Instruments) with a final resistance in the range of 80–120 MW. SMVs were visualized by phase-contrast microscopy with a Nikon or Zeiss inverted microscope. Signals were filtered at 5 kHz using the amplifier circuitry. Data were analyzed using pClamp 10.0 software (Axon Instruments). All population data were expressed as mean ± SEM. Membrane currents under different experimental conditions were assessed by measuring the peak membrane current (in pico-amperes) minus the baseline current. The baseline current was defined as a nonspecific electrode leak current. All current measurements were adjusted for the holding voltage assuming a linear current–voltage relationship: the resulting conductances are expressed in picosiemens according to the equation *G* = *V*/ΔI, where *G* is the conductance in picosiemens, *V* is the membrane holding voltage in millivolts, and ΔI is the peak membrane current in pico-amperes minus the baseline current in pico-amperes. Group data were quantified in terms of conductance.

### Mitochondrial membrane potential

Three-week-old WT and DJ-1^−/−^ cortical neurons were incubated in growth medium containing 5 nM TMRM (tetramethylrhodamine, methyl ester, a cell membrane permeable mitochondrial potential indicator) for 20 min at 37 °C CO_2_ incubator. Images were captured under standard conditions using Zeiss LSM 710 Duo confocal and the staining intensity data were analyzed using Zen software.

### Oxygen flux measurements

Oxygen uptake was measured in single neurons in culture in extracellular recording buffer using a sensitive oxygen electrode. Oxygen flux was recorded using a 2–4-μm-diameter electrode placed at 5 μm from the cell that was moved with a displacement of 10 μm in the *x*–*y* axes so that the oxygen-sensing electrode was positioned repeatedly closer to and farther from the cell. Signal extraction is performed by comparing near pole and far pole signal amplitudes obtained by excursion of the electrode repeatedly between the two poles (self-referencing configuration). In this manner, the effect of background, noise and drift are significantly reduced. The current detected at the two positions results in a differential current that is translated into O_2_ flux given the O_2_ concentration in the bath, the diffusion constant, and the distance of the excursion of the electrode between the two points.

### Quantitative real time RT-PCR

Total RNA was extracted from the midbrain culture neurons using RNeasy Plus Mini Kit according to the manufacturer’s protocol (Qiagen). Total RNA (1 µg) was reverse transcribed using Bio-Rad iScript first cDNA synthesis kit. Quantitation of mRNA levels was performed by TaqMan^®^ Gene Expression Assays (Thermo Fisher Scientific, USA). Analyses were calculated using the 2^−**ΔΔCT**^ method with β-actin as the normalizing endogenous control. The following probes were used:

**Table:** 

Name	Assay/Probe ID
hACTB	Hs01060665_g1
hATP5G1	Hs00829069_s1
hATP5G2	Hs01086654_g1
hATP5G3	Hs00909659_m1
hATP5B	Hs00969569_m1
hATP5O	Hs00426889_m1
mATP5G1	Mm02601566_g1
mATP5G2	Mm00848143_g1
mATP5G3	Mm01334541_g1
mActB	Mm02619580_g1
mATP5B	Mm01160389_g1

### Statistical analyses

Data in graphs were shown as mean ± SEM. For two group comparisons, paired or unpaired Student's *t*-tests (two-tailed) were used. For multiple comparisons, statistical analyses were performed with ANOVA. In some cases, data were normalized to control data before analysis (*P < 0.05; **P < 0.01; ***P < 0.001; ****P < 0.0001).

## Supplementary information

Supplemental Figures

Supplementary figure legends
