# Supplementary figures and images for: Parkinson’s disease protein DJ-1 regulates ATP synthase protein components to increase neuronal process outgrowth
Source: Cell Death Dis. 2019 Jun 13;10(6):469. doi: 10.1038/s41419-019-1679-x (PMC6565618; doi:10.1038/s41419-019-1679-x)

Fig. S1

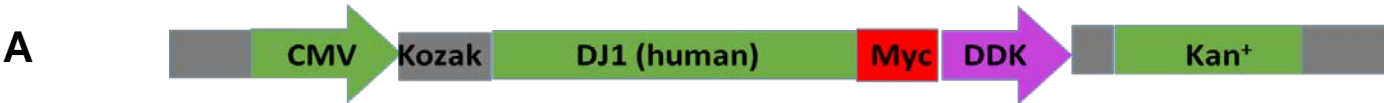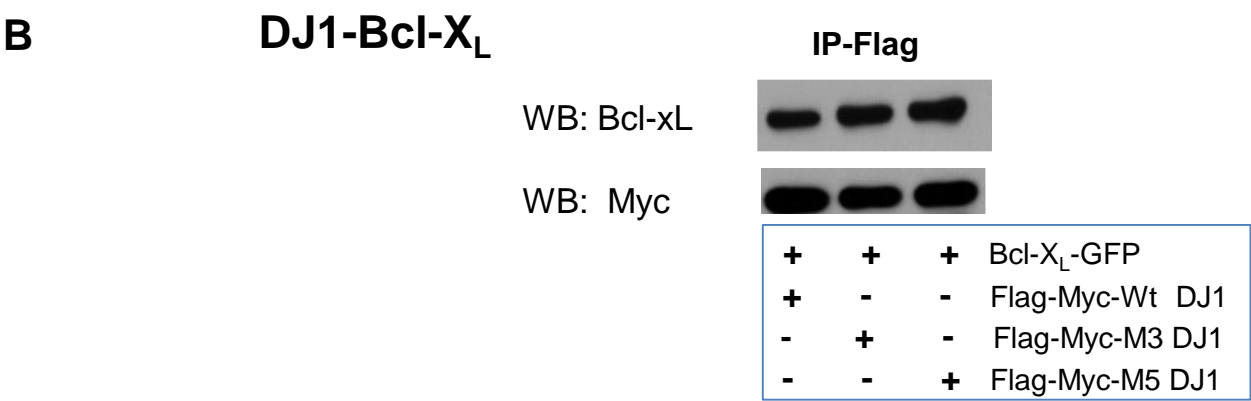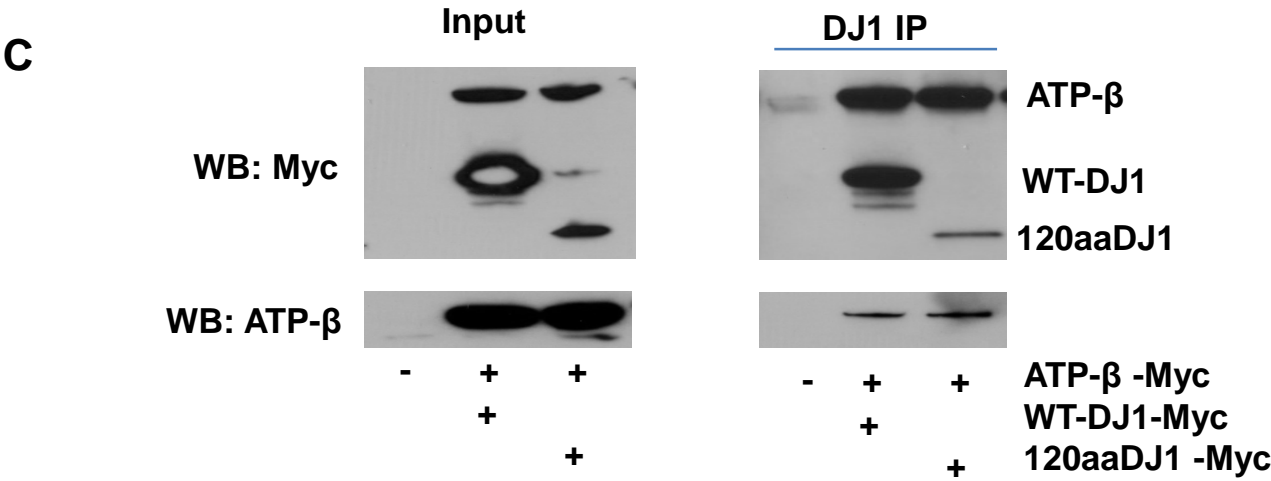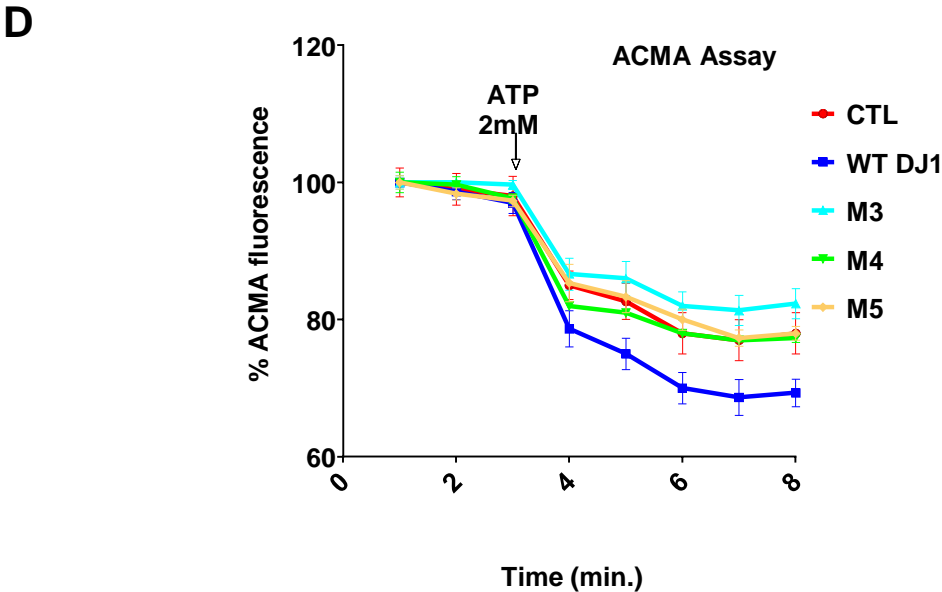

**Fig. S2**

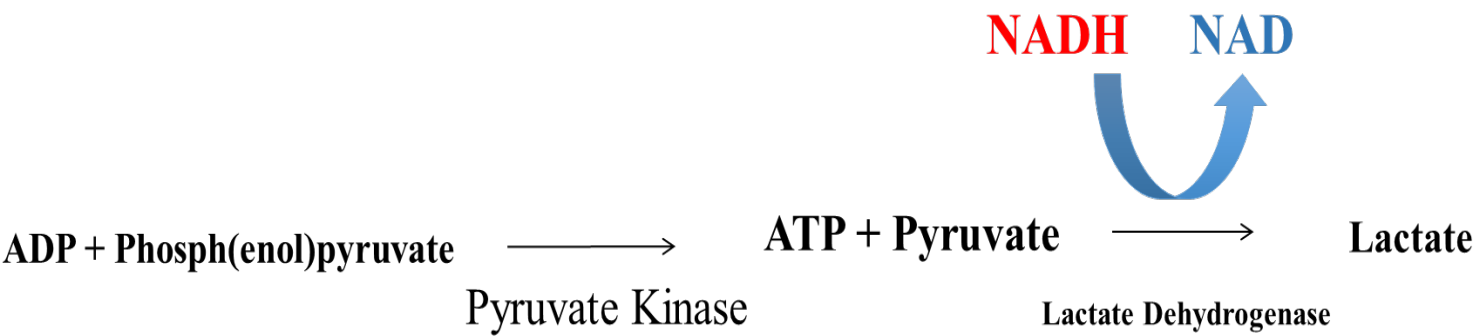

**Fig S3**

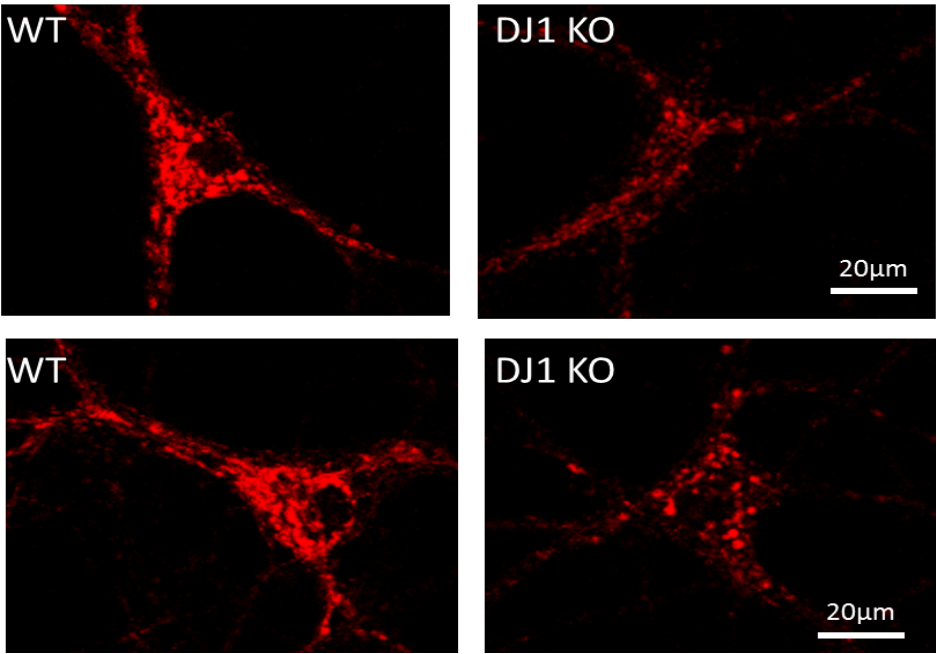

Fig S4

A

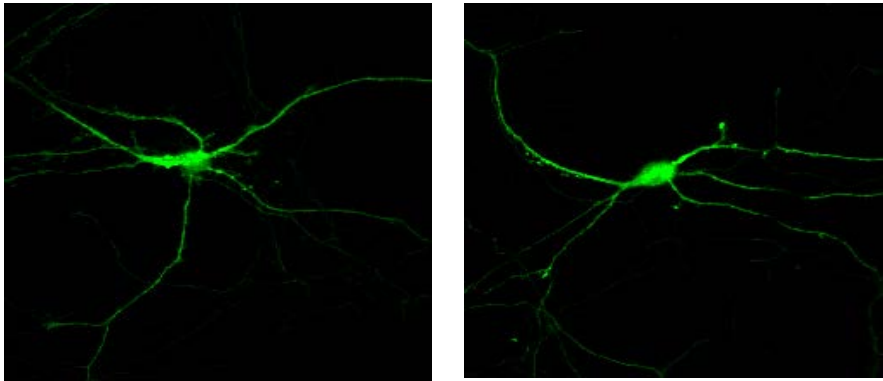

B

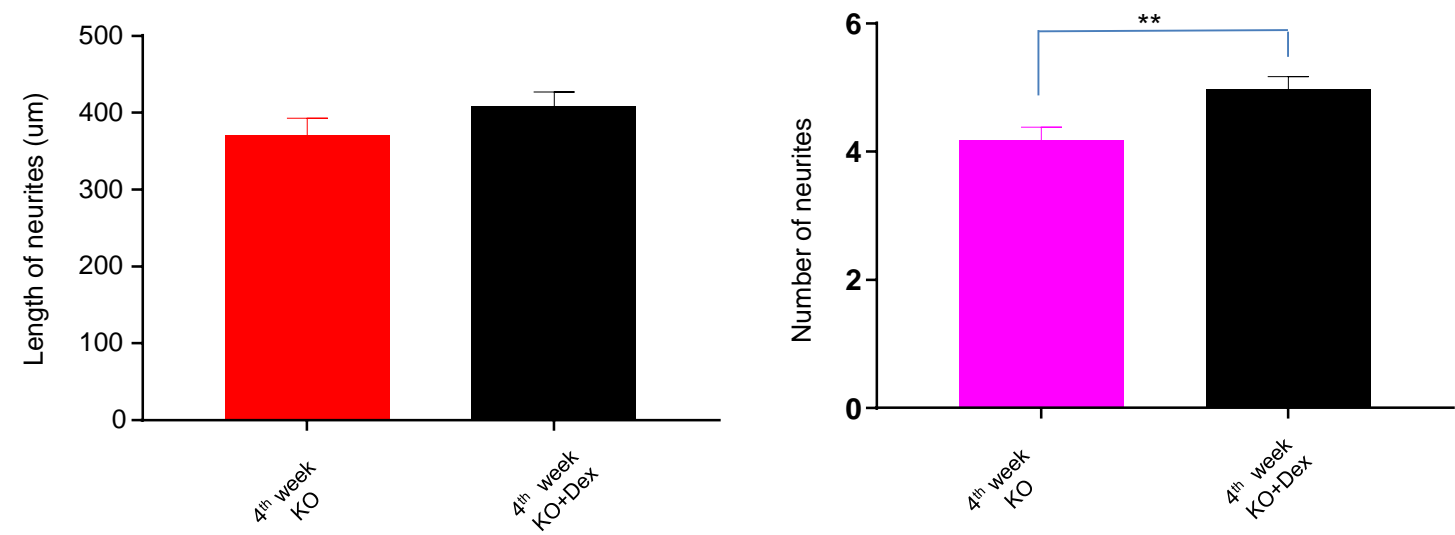

Supplement: Supplementary file 1 — Supplemental Figures [file 41419_2019_1679_MOESM1_ESM.pdf]
