# Supplementary material for: Parkinson’s disease protein DJ-1 regulates ATP synthase protein components to increase neuronal process outgrowth
Source: Cell Death Dis. 2019 Jun 13;10(6):469. doi: 10.1038/s41419-019-1679-x (PMC6565618; doi:10.1038/s41419-019-1679-x)
Supplement: Supplementary file 2 — Supplementary figure legends [file 41419_2019_1679_MOESM2_ESM.docx]

**Supplementary figure legends**

**Fig.S1.**

1. Vector map of the DJ-1-expressing plasmid.
2. Bcl-xL-GFP was co-expressed with Flag-Myc tagged tagged DJ-1 WT, M3 or M5 in HEK293 cells. Cell extracts were subjected to immunoprecipitation (IP) with Flag M2 agarose beads. Note that both WT and mutant DJ-1 interact with Bcl-xL.
3. Myc-tagged ATP synthase β subunit was co-transfected with full length or 120 amino acids N-terminal DJ-1. Cell extracts were subjected to immunoprecipitation of DJ-1, and western blot was performed with anti-Myc antibody or ATP synthase β subunit antibody. Full length DJ-1 or c-terminal deletion mutant DJ-1 both interact with ATP synthase β subunit.
4. The ability of SMVs to sequester H+ is improved by recombinant WT DJ-1 protein. Shown are ACMA fluorescence intensity changes in response to ATP in the presence of rat SMVs.

**Fig.S2.** Schematic of the ATP synthesis-NADH assay (see methods for details).

**Fig.S3.** TMRM staining of mouse hippocampal neurons; additional representative images are shown.

**Fig.S4.**

1. Representative immunocytochemistry images of dopaminergic neurons obtained using anti-Tyrosine Hydroxylase Antibody.
2. 10 µM Dex increases the number of neurites arising from the soma of DJ-1-/- TH+ neurons (right), but not the length of neurites (left).
